# Consideration of the Advisability of Athletics in Professional Colleges, with Special Reference to the University of Buffalo1Read before the faculties of Medicine, Law, Pharmacy and Dentistry of the University of Buffalo, December 17, 1904.

**Published:** 1905-02

**Authors:** Burton T. Simpson

**Affiliations:** Buffalo, N.Y.


### Consideration of the Advisability of Athletics in Professional Colleges, with Special Reference to the University of Buffalo.[1]

By BURTON T. SIMPSON, M. D., Buffalo, N. Y.

IN DISCUSSING this subject, my object is to state plain facts as they stand in the hope of bringing out a fair discussion of the subject, and a satisfactory solution of the problem.

I favor athletics here, but in view of the fact that our college closes so early in the spring, track athletics and baseball are out of the question; therefore I will only consider foot-ball, a game which is played in the fall, and the only form of athletics in which the University of Buffalo can participate.

Why should we have athletics in this university? This question naturally divides itself under two heads, first, the value to the university as an institution, and, second, the value to the students. It is upon these two propositions that I base my arguments.

In considering the value of athletics to the university, it will be well, first, to get some definite idea of what other colleges are doing in this direction.

There are 146 institutions of higher education represented by foot-ball teams. Of these 53 are purely technical colleges and it will interest us to know that 12 of these technical schools are medical colleges, among them some of the best in the country.

The past season Harvard University spent $80,000 to maintain athletics, and this is a fair figure for the other large institutions of her size. The University of Pennsylvania spent $25,000 for a gymnasium, and has invested $600,000 for an athletic field. I think you will agree with me that unless athletics were of decided benefit such sums would not be paid to foster sports. Not being satisfied that my conclusions upon the attitude of college officials would be convincing, I wrote to the presidents of some of the larger universities in the United States, asking for their individual opinions. Thirteen consider athletics beneficial to their institutions, while three only are negative. These figures give a higher percentage of negative replies than would be the case if I had a greater number of answers, for I find Prof. Dextar, of the University of Illinois, who has studied the question from the same standpoint, gives the percentage as 1 in 17, basing his figures upon 100 replies.

No one can deny public interest in foot-ball. One has only to look at local papers on the Sunday after a game to be convinced. The entire front page is usually given up to a description of the

1. Read before the faculties of Medicine, Law, Pharmacy and Dentistry of the University of Buffalo, December 17, 1904.

game, and usually there are photographs of the men who make-up the teams.

Let me give an illustration having local interest: a young man, who had quite a reputation in his own town as a high school foot-ball player, came to the University of Buffalo (and I might add he chose to come here because we had a foot-ball team) and entered the school of pharmacy. He played on our team, and succeeded in making the only score which won the Columbia game by a drop kick from the field. The papers of his native city, which are read by the people in the surrounding country for a radius of 25 miles, came out with a two-column article on its front page, with double head lines, to this effect: "University of Buffalo defeats Columbia University, 5-0. Carl Rice, who is studying pharmacy at the university makes the only score by a drop kick from the field."

Let us analyse the above statement: first, there is a University of Buffalo; second, it has a college of pharmacy; third, it has a foot-ball team; fourth, it is of such standing as to compete favorably with Columbia University.

What value does this have in attracting students to the university? Do they come to college to study foot-ball? Most certainly not; but whom do we get as students? I think you will agree with me that they are mostly high school graduates, at least that is what the records of the last four years show in this medical college. What is the average high school student's conception of a college? Usually, that it consists of a number of buildings, a body of students and an athletic field, where, even if he is not an athlete, he can go and cheer for the team which represents his college. This conception is formed, I think, from information derived from college men at home on their vacation, talking of the prowess of their college's athletes and athletic teams, and from newspapers.

Foot-ball is the most popular athletic sport we have. All small towns have teams, and nearly every high school in the country is represented by an eleven. I think it will surprise you somewhat, as it did me, to learn there are 57 organised teams in the City of Buffalo, which means at the lowest estimate 700 players.

That men have come to our college because we had a foot-ball team, I have letters to show, and I know of two men who went to other colleges this fall because we did not have athletics. I do not think, on the other hand, you can show me a man who went away because we did play foot-ball.

The *esprit de corps* among students and alumni is encouraged by athletics. It is a noticeable fact that the fraternal spirit of our students is emphasised by its absence, while true university spirit is absolutely lacking. That a foot-ball game where students can cheer for a common cause, standing upon common ground, is a most potent factor in the development of a closer college and university feeling, not even the most rabid antagonist to the game can gainsay. Once we develop this spirit in the students we need not worry about the future alumni.

An extract from the report of the committee on student organisation reads as follows regarding foot-ball: "It affords the only medium from which can be developed that *esprit de corps* which is at present the greatest need of the undergraduate body."

Chas. L. McKeehan, of the University of Pennsylvania writing upon "The university social problem," says: "I mean the great part athletics have played in developing a university spirit among students as we may distinguish from a departmental spirit;" while Jas. A. LeRoy, University of Michigan, in a similar article writes: "College sports themselves have done wonders in fostering an active interest among alumni and in binding them together."

A better standing with the people of Buffalo as reflected through the students would also result from properly administered athletics. I mean that through athletics those students who have a superfluous amount of animal spirits would have a legitimate outlet, and need not exhibit their natural effervescence by gate lifting, sign stealing, or other depredations upon private property. Some estimate may be formed of the feeling of the people of the city toward the university, by the fact that after a man has been in college a year he usually discards his U. B. pin which he so eagerly bought during his first month in college.

In this assembly of professional men I am sure not one undervalues the benefit of exercise. What do we offer our students along this line? I am quite sorry to say, nothing. The old adage says: "All work and no play makes Jack a dull boy," and if you look over the study schedules, you will be forced to admit that either students will be dull boys or else they must take the time when they ought to be studying or sleeping to get their recreation.

My first point, then, is exercise for students. Later I will suggest a scheme whereby all who wish may get the opportunity.

The value to the individual who plays the game may be best made clear by quoting men whose opinions should have great weight:

President Schurman, of Cornell University, in the president's report says: "College games, though liable to abuses, do beget in young men promptitude, quickness, alertness, hardihood, self-reliance, self-control, and the habit of coöperation, and self-surrender to authority." . . . The relaxation and discipline of healthy, manly sports are too valuable for American youth to forego; on the contrary they should under proper restraints, be encouraged in all seats of learning."

Rev. J. E. C. Welldon, head master at Harlow: "It was the instinct of sport which played the chief part in creating the British empire and that she owed her empire more to her sport than to her studies."

B. E. L. Richards, Professor of Mathematics, Yale, who has been a teacher for twenty-five years, in writing on the foot-ball situation says: "Foot-ball is one of the best forms of athletic sports that can be invented. No other sport is so beneficial." He sums up the benefits as: (1) body or physical—training health, strength; (2) mind—quickness of mind, as in signals, plays.

But great as are these benefits of the sport to the players in body and mind, they are not to be compared with its moral effect, which are: (1) courage, (2) self-control, (3) self-denial, (4) obedience, (5) discipline. The best teams at Yale have contained the most moral and religious men."

President Warefield, Lafayette College: "College athletics have done more to purify, dignify and elevate college life, than any influence brought to bear in the past quarter of a century. . . . . Foot-ball when properly played is a school of morals."

President Angell, University of Michigan: "I regard foot-ball as a valuable athletic game. It calls for and cultivates temperate and regular habits of living, vigor and agility of the body, quickness of perception, readiness of resource, manly courage, skill in planning, subinordination of the individual with coöperation of the team."

Hely H. Almond: "Foot-ball as a moral agent. When the complaint was made to a well known head-master that British boys talked far too much about foot-ball and cricket he answered, 'And what do French boys talk about?' "

C. F. Twing, LL.D., President Western Reserve University: "Before and above these evils, I would emphasise its (foot-ball) function in developing the gentleman of ethical character and conduct. For foot-ball represents the inexorable, it teaches the value of the positive, illustrates the worth of a compelling interest, it promotes self-discovery and disciplines self-restraint."

With this array of quotations from such prominent educators to which many could be added, we ought, at least to see some value to the men who participate.

ARGUMENTS AGAINST FOOT-BALL.

As near as I can gather the arguments against foot-ball at our university are as follows:

A man who is pursuing the study of a profession has no time for athletics.

The liability to accidents.

The student body contracts debts which remaining unpaid give the university a bad name.

I grant that a man studying medicine cannot learn all he should know in four years; neither do I think he could in ten years. On the other hand, a man can do better work throughout the winter for having exercised during the fall. In regard to time—foot-ball requires only two hours a day, from 4 to 6, during the first two months of college, time when no one really gets down to hard work, and this only applies to about twenty men in the whole university. A man misses no work that he is not able to make up, and in the majority of cases he can so arrange his schedule that in any event he will not miss many classes.

That a man can play foot-ball and keep up his work is clearly shown by the records of the men on teams in past years. Exactly as do the records of non-foot-ball men show, I might observe that the men whose records are poor, were only substitutes, and although physically competent to play never made the team, because they lacked the grit.

The matter of doing good work is easily taken care of, for nearly all colleges require that a man must obtain a certain percentage in his work or else he is dropped from the squad. In some colleges, notably Yale, a foot-ball man is required to obtain a higher average percentage than the rest of the men in his class. In regard to technical students playing foot-ball, I might call attention to the fact that Torry, captain of University of Pennsylvania, last year's champions, is a medical student, while Costella, Cornell's captain is an electrical engineer. On last year's Michigan championship team four men were mechanical engineers, while six were law students. The fact that fifty-three technical schools have foot-ball teams shows that a man has time if he has a mind to work. I wish to quote Walter Camp, a professor at Yale, and the recognised authority on foot-ball matters. In a letter to a mother inquiring if she should let her son play foot-ball, he said: "If Edward is a 'high stand' man it is quite certain his standing will suffer somewhat on account of the time and attention devoted to the sport. If he be a 'low stand' man this would hardly be the case, owing to the effort and the pressure brought to bear upon him by his captain and his fellows to keep up the average which is necessary to secure the permission of the faculty for him to take part."

In three years' experience as coach of Masten Park High School team, where the study average required is 80 per cent. I have never been compelled to drop a man. The knowledge of the fact that poor scholarship is not compatible with being a member of the team is a great stimulus to do good work in school.

In fourteen years' experience in playing and coaching foot-ball, I have seen but five accidents which might be called serious, one fractured clavicle, three fractured femurs and one fractured rib.

By careful estimation I find that the very lowest figures show that 46,000 young men are playing foot-ball each fall. The report of this year's deaths and accidents are as follows: eleven deaths 2-100 of 1 per cent.; 121 injured 2-10 of 1 per cent.

In looking over the statistics I find that only one trained athlete died, and that with but one exception none of the injuries that occurred on the big college teams, where the men are properly trained, incapacitated the players from keeping up their work.

In looking up statistics in regard to casualties I was struck by the tameness of foot-ball, as compared with some other sports. For instance, hunting. In the north woods of Wisconsin and northern Michigan, forty-two men were killed or died of their wounds, while twenty were wounded in the short season of twenty open days.

The matter of contracting debts is easily adjusted if the team is properly managed. The manager should be a graduate, and one who is responsible to the faculty. A good team more than pays all its expenses. We may get some idea of this by reading the report of the graduate manager of the Harvard Athletic Association, which shows that athletics made a profit this year of $33,051. Foot-ball alone made $57,223, the losses and expenses in other branches bringing the profit down to the figures mentioned.

I wish now to give some conception of the game and the manner in which the men train. In the first place it is the most manly and scientific athletic game at present known. A man to play must be in the pink of condition; also, he must be gentlemanly and intelligent. He must train faithfully and the essential points of training are as follows: smoking, drinking and all excesses are absolutely forbidden. A man's diet must be plain and wholesome. He should maintain regular hours and get a normal amount of sleep. In the early part of the season he is taught how to fall on the ball, kick, run properly and is given such light work as will get his body in physical condition, so that the liability to injury is reduced to a minimum.

The game is played by two teams of eleven men each, and the fundamental idea is to place the ball behind the opponents' goal line. The side having the ball is allowed three chances to advance the ball at least five yards; in case of failure to do so it goes to the opposing team.

In order to advance the ball we must have team work, and this requires that every man has his particular place to fill in every play; to accomplish this the plays are run by signals and as there are some fifty-five different plays and a signal for each, it is necessary for a man to have some brains in order to play.

The foot-ball field is a test of self-control, for a good player never loses his temper. He must be cool. It teaches him self-confidence, ability to take advantage of situations. For frequently the play as started will be blocked by the opponents and then the man with the ball must pick and use the most advantageous way through or be thrown for a loss.

In looking over the history of our experience with foot-ball, I can see many things that have been detrimental to all concerned, and some things which could have been improved upon. In the first place, I hardly think the faculty have exhibited enough interest in the sports of the college. They have failed to require men to keep up in their work or else drop playing foot-ball. They have allowed men to play on some of our teams who were not registered in any college of the university, and they have not attended the games themselves, thereby encouraging men to participate.

The first two conditions admit of no argument. The third does, and the time honored doctor's excuse is given: "I don't have the time." Well, that is true, but time could be taken. When I was in college one of our most prominent professors, and a very busy man, said there would be no class on the following Saturday as he was going down to New Haven to see his Alma Mater whip Harvard.

What do you think would happen to foot-ball in this city if the members of the faculties of the University of Buffalo and their families were to occupy boxes at our games? It would stimulate foot-ball to such an extent that each game would be a social event in Buffalo. I can't find fault with the faculties as far as contributing is concerned, for they have been very generous, but what is needed to stir up this movement is a knowledge among the students that the authorities are favorable and mean to support it by their presence.

In the past the team has not been properly managed. Besides this, men have been allowed to play on the team who were not bona fide students. The team, from lack of support, was allowed to struggle along, without proper coaching and outfitting, getting from bad to worse, and I for one was glad to see it come to an end.

A good foot-ball team will earn money. A prominent lawyer in this city, who has had experience in managing college teams will manage our team for half of the net receipts and he told me he would expect to make $2,500 each season.

If we have a team, let us have a first-class one, properly coached and managed, well supported and an honor to the university.

The chief objection to foot-ball and kindred games carried on in the colleges at present, is the amount of time and money used on a small body of men, who really do not need the physical training, while the vast body of men and especially the book-worms, who really do need the exercise and recreating, do not participate. I admit this point, and on its face it looks like a very strong argument; but there is another side to the question. It is decidedly advantageous to the few who do participate and it pays for all the other varieties of gymnasium and athletic sports.

As I have mentioned before in this paper, the university does not offer to the students anything in the line of physical training; probably because there is no appropriation for it, for I am sure that it is not neglected because the authorities underestimate its worth.

My plan is to use foot-ball for the benefit of those who participate, and to make money which could be used to provide athletic opportunities for the rest of the students.

Last year with our poor team, which played five games here, and with very ordinary teams, we paid the Athletic Field, simply for the privilege of playing there, 30 per cent. of the gross receipts, and this amounted to $839.00. This could easily, with a good team well advertised, be tripled. Therefore I think even with this money we could run a field of our own and make it the campus for the entire body of students, where we could stimulate interclass foot-ball games, intercollege tug-of-war teams, a track for men so inclined, and a place to play a little base-ball in the early fall. With the first money earned, I would suggest that we put a series of hand-ball courts between the medical and dental colleges, a game which is of great value physically, requires no apparatus, and can be built for a small sum of money. Students could go there when they had a vacant hour during the day, and take sufficient exercise to keep their bodies in excellent condition. Later we could increase the size of the building, so as to include some gymnasium apparatus, bowling alleys and possibly baths. Therefore, if we can make foot-ball pay and thus provide facilities of exercise for the body of students, I think the strength of the point that the game only benefits the few is largely lost.

151 FRONT AVENUE.

### DISCUSSION.

Mr. W. B. WRIGHT, of law, agreed, in the main, with Dr. Simpson and declared that a poor team was worse than none at all. If the university has a football eleven it must be a winning team.

REV. R. B. ADAMS was strongly in favor of a team for the university. He recalled the fact that the cadets at West Point had only one hour a day for practice, yet were able to put up a winning team. Out of the 447 students at the University of Buffalo he thought it should be easy to secure 25 men to play on the team.

Mr. VOGHT, principal of the Central High School, was heartily in favor of games and outdoor sports.

Dr. DELANCEY ROCHESTER was unequivocally opposed to football, but favored outdoor sports. He said the mortality and injury rate were too high for the game as a game; the exercise was too severe.

Dr. M. D. MANN did not believe football could be played well by a university team.

Dr. CHARLES CARY was heartily in favor of the plan and promised his support in the future as freely as it had been given in the past.

Mr. JOHN LORD O'BRIEN'S opinion was that the sport had never been properly regulated; that a university spirit was necessary and the best way to develop it was by means of football. The game should have at least one fair trial.

Mr. ADELBERT MOOT had been opposed to the game on account of its professionalism; but if this could be removed and the game kept free from it he was in favor of a university team.

Dr. JAMES A. GIBSON, professor of anatomy, knew of several good men who had left the medical school because of the lack of university spirit. He was heartily in favor of a team.

Dr. GREGORY, of the pharmacy college, thought the whole question hinged on proper management. With that solved there should be a team.

Dr. SNOW, of the dental school, is personally opposed to football; but if it is decided to have a team he would see that the dental department was represented.

Dr. LUCIEN HOWE expressed himself in favor of athletics and said the men should have a chance to rub elbows.

Dr. HOOVER said the 'varsity team had originally been started in good shape and under honest management; laxity in business affairs, the introduction of professionalism and general apathy on the part of the faculty had caused the game to fall into disfavor. Dr. Otto spoke along the same lines.

Following the discussion a vote was taken, on the proposition to secure an expression of opinion, which resulted in 29 in favor of football and 4 against.

